# Identification and Functional Characterization of Novel Phosphorylation Sites in TAK1-Binding Protein (TAB) 1

**DOI:** 10.1371/journal.pone.0029256

**Published:** 2011-12-22

**Authors:** Alexander Wolf, Knut Beuerlein, Christoph Eckart, Hendrik Weiser, Beate Dickkopf, Helmut Müller, Hiroaki Sakurai, Michael Kracht

**Affiliations:** 1 Rudolf-Buchheim-Institute of Pharmacology, Justus-Liebig-University Giessen, Giessen, Germany; 2 Division of Pathogenic Biochemistry, Institute of Natural Medicine, University of Toyama, Toyama, Japan; Wake Forest University, School of Medicine, United States of America

## Abstract

TAB1 was defined as a regulatory subunit of the protein kinase TAK1, which functions upstream in the pathways activated by interleukin (IL)-1, tumor necrosis factor (TNF), toll-like receptors (TLRs) and stressors. However, TAB1 also functions in the p38 MAPK pathway downstream of TAK1. We identified amino acids (aa) 452/453 and 456/457 of TAB1 as novel sites phosphorylated by TAK1 as well as by p38 MAPK in intact cells as well as *in vitro*. Serines 452/453 and 456/457 were phosphorylated upon phosphatase blockade by calyculin A, or in response to IL-1 or translational stressors such as anisomycin and sorbitol. Deletion or phospho-mimetic mutations of aa 452–457 of TAB1 retain TAB1 and p38 MAPK in the cytoplasm. The TAB1 mutant lacking aa 452–457 decreases TAB1-dependent phosphorylation of p38 MAPK. It also enhances TAB1-dependent CCL5 secretion in response to IL-1 and increases activity of a post-transcriptional reporter gene, which contains the *CCL5* 3′ untranslated region. These data suggest a complex role of aa 452–457 of TAB1 in controlling p38 MAPK activity and subcellular localization and implicate these residues in TAK1- or p38 MAPK-dependent post-transcriptional control of gene expression.

## Introduction

A complex interplay of protein kinases and their phosphorylated substrates regulates major aspects of immune and stress responses [1]. One of these kinases is TGFβ-activated protein kinase (TAK)-1 which is activated by proinflammatory cytokines (IL-1, TNF, IL-18), pathogens, RANKL, stresses and during T- and B-cell activation and thus represents a prototypic central effector acting upstream of NF-κB, JNK and p38 MAPK signaling pathways [2]–[6].

TAK1 activation is tightly controlled by reversible phosphorylations, non-degradative ubiquitination and by protein:protein interactions. The latter include interactions with TAK1-binding proteins (TAB) 1–3 which all have been shown to participate in TAK1 activation. Hence, TAB1–3 can be viewed as crucial regulatory subunits of the active TAK1 kinase complex [7]–[12]. In TAB2 and TAB3, C-terminal Zn-finger motifs provide a docking surface for K63-linked ubiquitin chains which are conjugated by E3-ligases such as TRAF6 or TRAF2 to various signaling intermediates after activation by innate immune receptors. These covalently attached ubiquitin-chains recruit TAK1 in complex with TAB2 or TAB3 to IL-1, TNF or TLR receptors [13], [14].

The TAB1 subunit is also present in TAK1/TAB2-polyubiquinated immunoprecipitated protein complexes after IL-1 stimulation [8], [15]. However, unlike TAB2 or TAB3 it apparently does not serve to direct TAK1 to receptors of the immune response [10]. Instead, a regulatory domain contained in amino acids 437–504 of TAB1 binds to TAK1 and is fully sufficient to activate ectopically expressed TAK1 suggesting that the primary role of TAB1 is the regulation of TAK1 catalytic activity [16]–[19].

In addition to TAK1, TAB1 interacts with p38 MAPK and activates its autophosphorylation by an allosteric mechanism. TAB1-mediated p38 MAPK autoactivation occurs independent from all three p38 MAPK-activating kinases (MKK3, MKK6, MKK4) but accounts for only a small portion of overall p38 MAPK activity in a cell-and stimulus-dependent manner [20]–[24].

**Figure 1:**
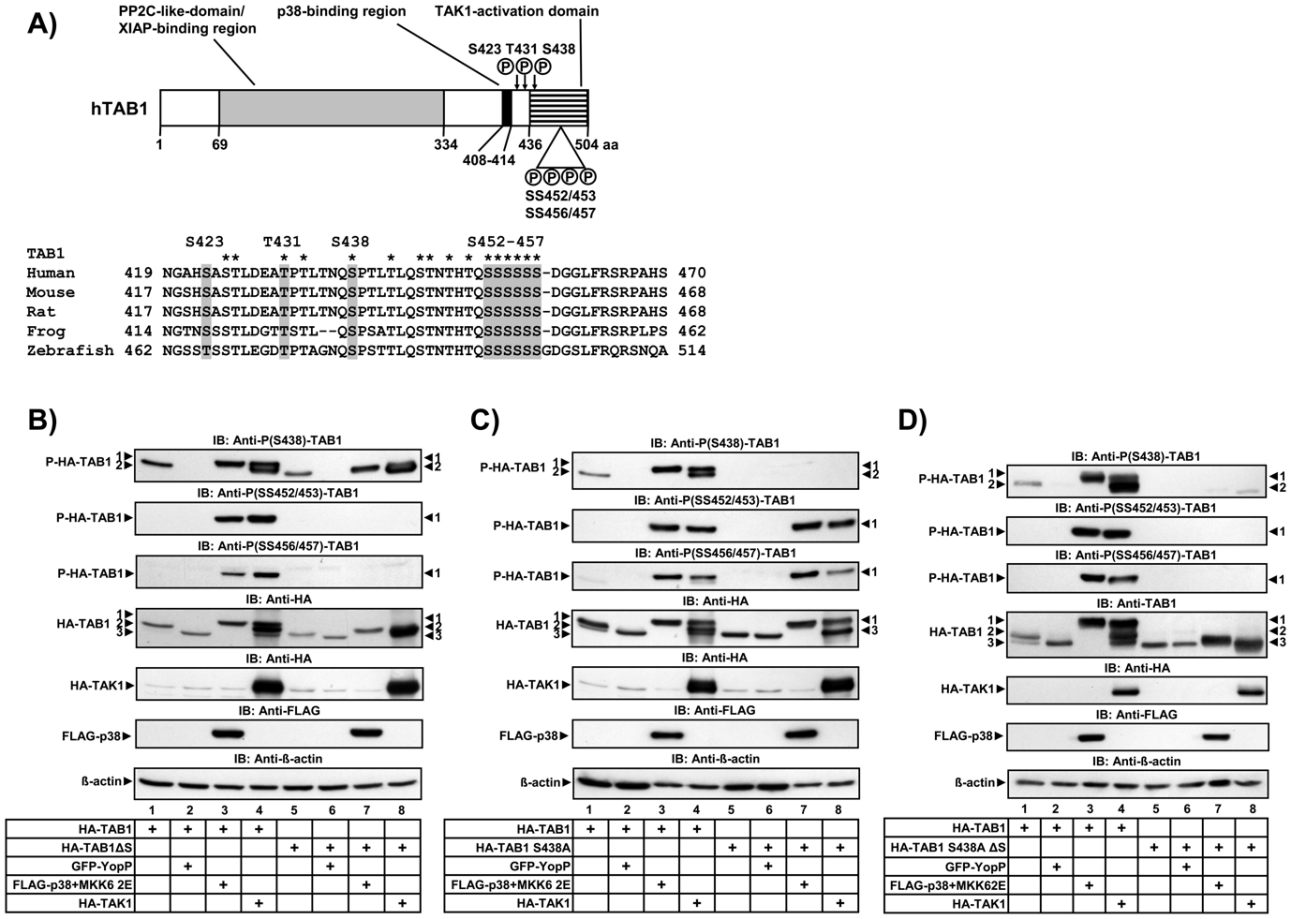
Identification of new phosphorylation sites in TAB1. A) Upper panel: schematic structure of TAB1 indicating functional domains and known phosphorylation sites (S423, T431, S438) as well as the new sites described in this study (SS452/453, SS456/457). Lower panel: alignment of part of the TAK1 activation domain of TAB1. Conserved potential phosphorylation sites are indicated by asterisks, phospho amino acids analyzed in this study are shaded gray. B)–D) HEK293IL-1R cells were transiently transfected with expression vectors for HA-TAB1 wild type or versions in which S438 was mutated to alanine and/or aa 452–457 were deleted (TAB1ΔS) alone or in combination with GFP-YopP, FLAG-p38 MAPK plus MKK62E or HA-TAK1 as indicated. 24 h later cells were lysed followed by immunoblotting (IB) to detect HA-TAK1, FLAG-p38 MAPK or HA-TAB1 antigens and the phosphorylated forms of TAB1 using the indicated antibodies. Equal loading of lanes was confirmed using anti ß-actin antibodies. Black arrowheads indicate the three forms of TAB1 (numbered 1–3) with different mobility upon SDS-PAGE as previously described by us [15].

As illustrated in the upper panel of Fig. 1A, three functional domains in TAB1 have been defined resembling the aforementioned TAK1 C-terminal activation domain [16], [17], a p38 MAPK interaction domain [22], [24] and a pseudophosphatase domain [25].

By mass spectrometry and by phospho-site specific antibodies, TAB1 was shown to be phosphorylated at S423, T431 and S438 by ERK1, p38 MAPK or JNK [20], [26]. Inhibition of these kinases [20], [26] or ectopic expression of a dominant negative TAB1 ST423/431AA mutant [6] revealed a role of these residues in controlling TAK1 enzymatic activity by a negative feedback mechanism that inhibits TAK1-activation [6], [20], [26]. In addition, inactivation of TAK1 can result from dephosphorylation by the serine/threonine phosphatases PP2C, PP6 and calcineurin [27]–[29] or from inhibition by bacterial virulence factors such as YopP [15]. All these observations point to a complex but only partially understood array of regulatory mechanisms that shapes the functions of the TAB1–3 proteins in the TAK1 and p38 MAPK pathways.

In particular, the physiological role of TAB1 is still enigmatic. While ablation in mice or RNAi-mediated suppression of TAB1 has no effect on IL-1-, TNF-, or TLR-induced activation of NF-κB, JNK, or p38 MAPK signaling pathways, suppression of TAK1 abolishes these signals [2], [3], [6], [26], [30]–[33]. A recent report using reconstituted TAB1-deficient fibroblasts suggested that TAB1 functions specifically in osmotic stress-induced TAK1 and subsequent JNK activation providing first evidence for a highly selective function of TAB1 in TAK1-signaling [34].

In previous experiments aimed at investigating the effects of activated p38 MAPK or TAK1 on TAB1 we have described three different posttranslationally modified forms of TAB1 that can be distinguished based on mobility shifts upon SDS-PAGE [15]. Here, we report the identification of novel TAK1- and p38 MAPK-mediated phosphorylation sites underlying these shifts. We also present evidence suggesting that the serine cluster containing these phosphorylation sites in TAB1 is involved in regulation of TAB1 and p38 MAPK subcellular localization and affects post-transcriptional gene expression.

## Results

### Identification of novel phosphorylation sites in TAB1 at amino acids 452/453 and 456/457

In addition to the already described S423, T431 and S438 residues, the C-terminal part of TAB1 contains several conserved serine/threonine residues which may also be subject to regulatory phosphorylations (Fig. 1A, lower panel). Initial experiments using mutated TAB1 versions revealed that the major TAK1- or p38 MAPK-inducible shifts of TAB1 occurred independent of S423/T431/S438 (Fig. S1A). As exemplified for S425, individual mutation of some of the other conserved sites in the C-terminal part of TAB1 had no effects on TAB1 mobility shifts (Fig. S1B, compare lanes 5–8 with lanes 1–4).

In contrast, deletion of six serines comprising aa 452–457 (TAB1ΔS) abolished TAB1 form 1 in response to both, overexpressed MKK62E/p38 MAPK (Fig. 1B, lane 7) or overexpressed TAB1/TAK1 (Fig. 1B, lane 8) despite normal phosphorylation of S438 as assessed by a phospho-specific antibody (Fig. 1B). Of note, under these activated conditions, the deletion mutant TAB1ΔS migrated faster and mainly occurred as mobility form 2 (Fig. 1B, lanes 7, 8). As previously published by our group [15], YopP (Fig. 1B, lane 6) shifted TAB1ΔS further downwards to form 3 which is explained by YopP affecting both, phosphorylation of aa 452–457 as well as the previously characterized p38 MAPK site S438.

In order to identify which of the six serines were phosphorylated, we generated phospho-specific antibodies against di-phosphorylated peptides containing either SS452/453 or SS456/457. A careful mutational analysis revealed the specificity of these antibodies for their respective residues (Fig. S2). As shown in Fig. 1B–D, both, activated p38 MAPK and TAK1 induced the phosphorylation at SS452/453 and SS456/457, an effect which was abolished by deleting the serine cluster of aa 452–457 in the TAB1ΔS variant (Fig. 1B). Moreover, phosphorylation at SS452/453 or SS456/457 under these conditions was independent from mutation of the p38 MAPK site S438 (Fig. 1C). However, mutation of both, S438 and aa 452–457 abolished all major TAB1 shifts suggesting that these amino acids are the prevalent sites of TAB1 modifications in intact cells (Fig. 1D).

**Figure 2:**
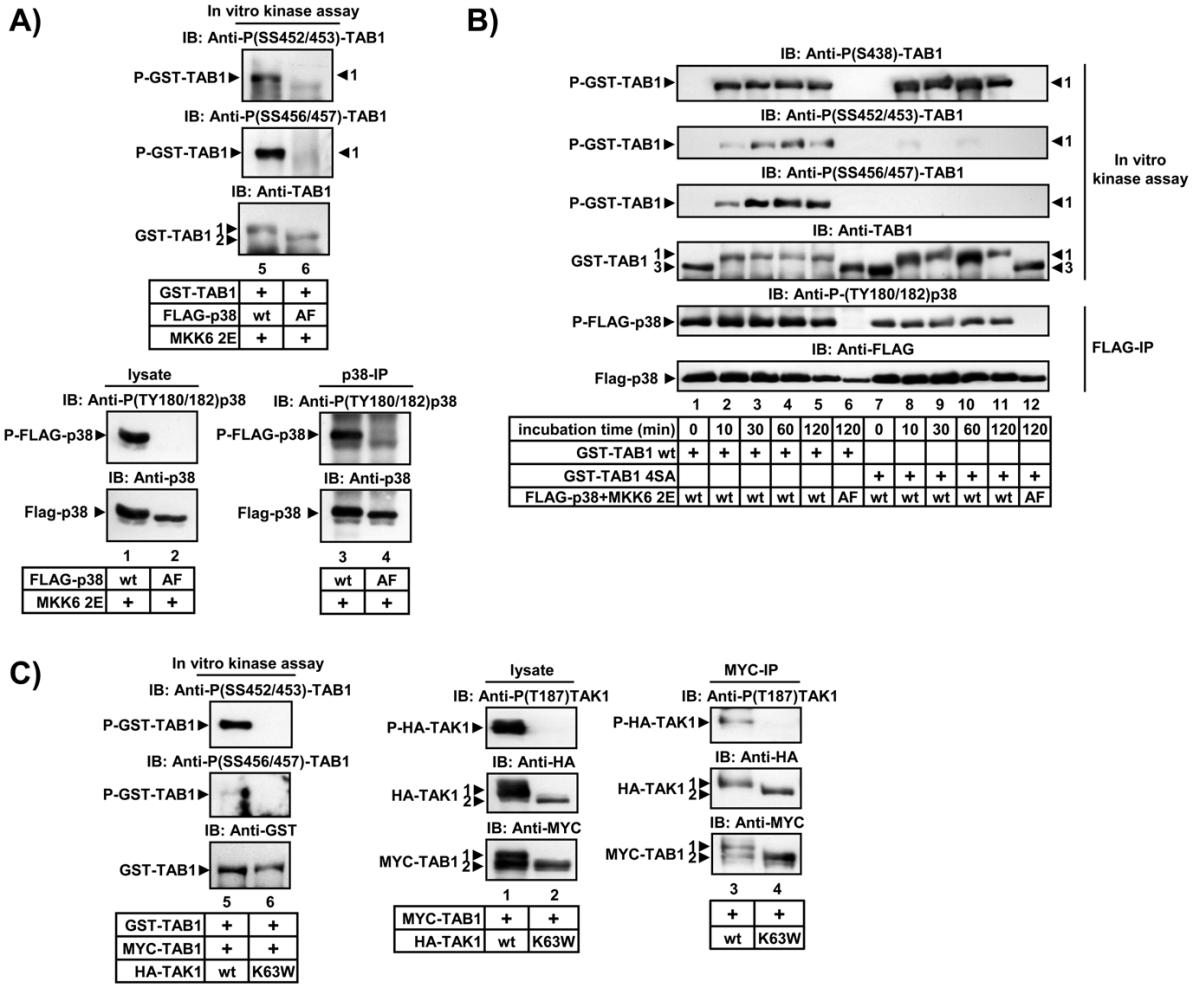
Active p38 MAPK and TAK1 phosphorylate SS452/453 and SS456/457 of TAB1 *in vitro*. A, B) HEK293IL-1R cells were transiently transfected with expression vectors for FLAG-p38 MAPK, inactive FLAG-p38 MAPK (T180A/Y182F, AF) or MKK62E as indicated. In C) cells were transfected with HA-TAK1, or inactive HA-TAK1 K63W plus MYC-TAB1 in the indicated combinations. 24 h later cells were lysed and used to isolate activated p38 MAPK (A, B) or TAK1 (C) by immunoprecipitation (IP). Activated p38 MAPK or TAK1 were incubated with recombinant GST-TAB1 and ATP as substrates. After 30 min, *in vitro* kinase reactions were terminated by separating GST-TAB1 with GSH-sepharose (A, C). Phosphorylation of GST-TAB1 *in vitro* was analyzed by immunoblotting using phospho-SS452/453, phospho-SS456/457 and phospho S438 specific antibodies. B) A similar experiment as in A) was performed using a GST-TAB1 SSSS452/453/456/457AAAA (GST-TAB1 4SA) mutant in parallel. The assay also demonstrates the kinetic of aa 452–457 phosphorylation by p38 MAPK *in vitro*. Equal immunopurification of kinases and of recovery of GST-TAB1 was validated by immunoblotting of lysates, immunoprecipitates or GST-TAB1 purified from reaction mixtures by the indicated antibodies. Comparable amounts of inactive p38 MAPK (AF) and TAK1 (K63W) were used in parallel to demonstrate the specificity of the kinase reactions.

Using immunopurified active p38 MAPK we also found that p38 phosphorylates recombinant GST-TAB1 (Fig. 2A, B) but not a GST-TAB1 SSSS452/453/456/457AAAA mutant *in vitro* with a kinetic similar to that of the already known p38 MAPK site S438 (Fig. 2B). A similar result was obtained for activated TAK1 (Fig. 2C). These assays further established the specificity of the phospho-specific antibodies and revealed SS452/453 and SS456/457 as direct novel target sites for both, p38 MAPK and TAK1.

### Phosphorylation of TAB1 at amino acids 452–457 is regulated by stress stimuli and cytokines

**Figure 3:**
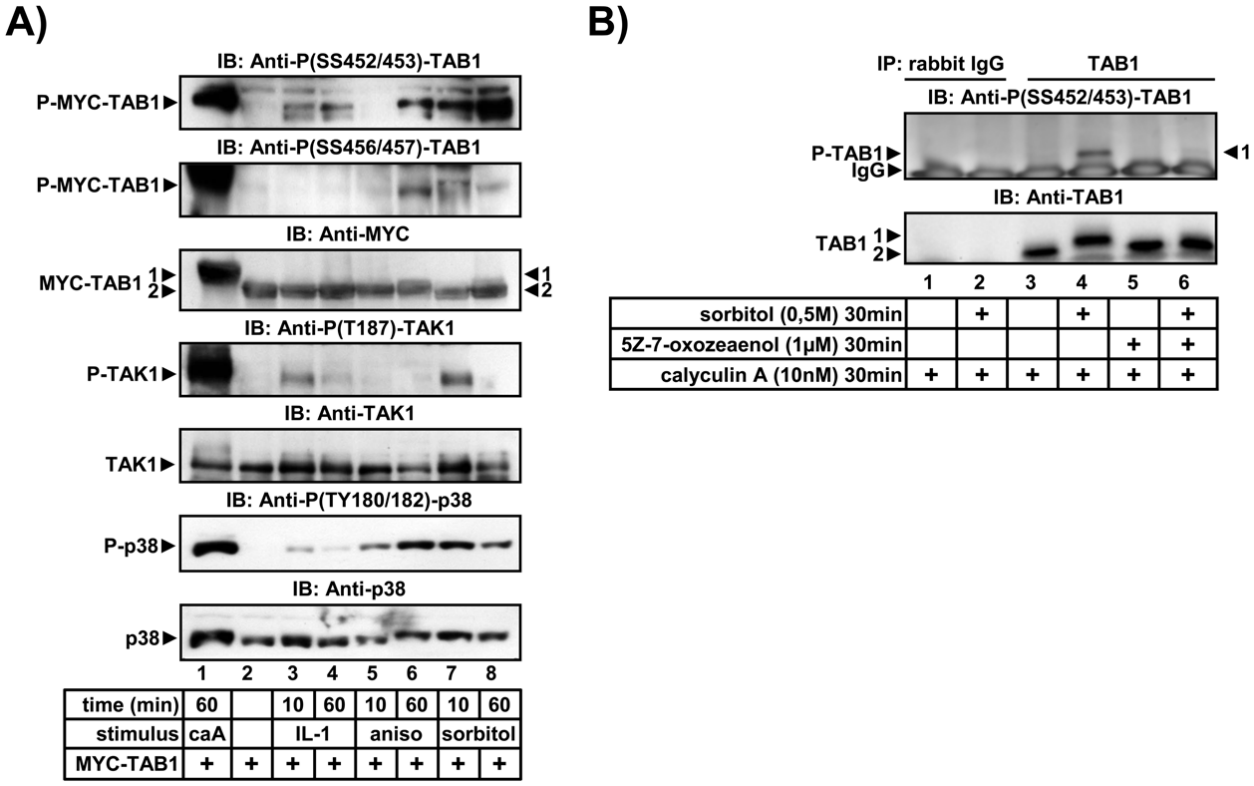
Phosphorylation of TAB1 at SS452/453 or SS456/457 in response to IL-1 or stress-stimuli. A) HEK293IL-1R cells were transiently transfected with an expression vector for MYC-TAB1. 24 h later, cells were treated with 40 nM calyculin A, 10 ng/ml IL-1α, 10 µg/ml anisomycin or 0.5 M sorbitol for the indicated times or were left untreated. Lysates were subjected to immunoblotting (IB) to detect phosphorylation of TAB1 using phospho-SS452/453-TAB1 or phospho-SS456/457-TAB1-specific antibodies. Phosphorylation of p38 MAPK at T180/Y182 and of TAK1 at T187 and equal loading of MYC-TAB1, TAK1 and p38 MAPK were analyzed using the indicated antibodies. B) HEK293IL-1R cells were treated for 30 min with 10 nM calyculin A in all samples and with the TAK1 inhibitor 5Z-7-oxozeaenol (1 µM) as indicated followed by sorbitol (0.5 M) for 30 min, by sorbitol alone or were left untreated. Thereafter, cells were lysed, proteins were immunoprecipitated with anti-TAB1 or control (IgG) antibodies and TAB1 or phospho-TAB1 were detected by the indicated antibodies.

We then tried to identify the stimuli which regulate SS452/453 and SS456/457 phosphorylation. It has been previously noticed that activation of TAK1 by proinflammatory cytokines such as IL-1 is transient, because it's catalytic activity is rapidly downregulated by serine/threonine phosphatases such as PP6 [28], PP2C isoforms [27], [35], [36] or calcineurin [29]. In line with the dependency of activation of the TAK1 complex on reversible phosphorylation, the strongest condition that induced phosphorylation of TAB1 at SS452/453 or SS456/457 was treatment of cells with the phosphatase inhibitor calyculin A (Fig. 3A, lane 1). SS452/453 were also phosphorylated in response to IL-1, anisomycin or sorbitol treatment, whereas detectable SS456/457 phosphorylation was only observed with anisomycin or sorbitol (Fig. 3A). In these experiments intracellular TAB1 levels were increased by overexpression of MYC-TAB1 to increase sensitivity of detection. All conditions shown in Fig. 3A also activated p38 MAPK to varying degrees which correlated with the level of TAB1 phosphorylation.

We also found that phosphorylation of endogenous TAB1 at SS452/453 could be detected by immunoprecipitating TAB1 from sorbitol-stimulated cells. This effect was suppressed by the TAK1 inhibitor 5Z-7-oxozeaenol confirming that these sites are regulated by a TAK1-dependent pathway in (patho)physiological settings (Fig. 3B). However, phosphorylation of endogenous TAB1 was only measureable when low concentrations of phosphatase inhibitors were added to the cells prior to cell lysis and by using the strongest TAK1 stimulus sorbitol suggesting that phosphorylation of TAB1 at aa 452–457 is very low abundant.

### Amino acids 452–457 of TAB1 are not involved in TAK1 activation

Since the best defined function of TAB1 is the activation of TAK1 in a co-expression system, we transiently co-transfected wild type TAB1 or the TAB1ΔS mutant together with TAK1. As shown in Fig. S3, in HEK293IL-1R cells, TAB1ΔS had no effect on *in vitro* kinase activity of immunoprecipitated TAK1:TAB1 complexes (Fig. S3A) and only very little effect on TAK1:TAB1 activation of IL-8 transcription (Fig. S3B), a gene whose expression is controlled by the TAK1 pathway [11]. Stable reconstitution of MYC-TAB1 in TAB1-deficient mouse embryonic fibroblasts partially restored *in vitro* kinase activity of immunoprecipitated TAK1 complex in response to calyculin A treatment compared to wild type cells (Fig. S3C). However, this effect was not influenced by reintroducing the S423, T431, S438 or the ΔS mutants in the TAB1-deficient background (Fig. S3C). We also tested if aa 452–457 were involved in ubiquitination of TAK1 and of TAB1 itself under conditions previously described by us [15]. However, deletion of aa 452–457 did not affect ubiquitination of either TAK1 or TAB1 (Fig. S3D). Collectively, these data suggested that aa 452–457 did not disturb correct folding of the minimal TAK1-activation domain comprising amino acids 480–504 [17] and did not play a role in TAK1 activation.

### An active mutant of p38 MAPK phosphorylates TAB1 at aa 452/453 in the cytoplasm

We, therefore, tested a potential role of aa 452–457 in activation and localization of p38 MAPK based on reports of a direct interaction between p38 MAPK with TAB1 [20]–[22], [24], [37].

**Figure 4:**
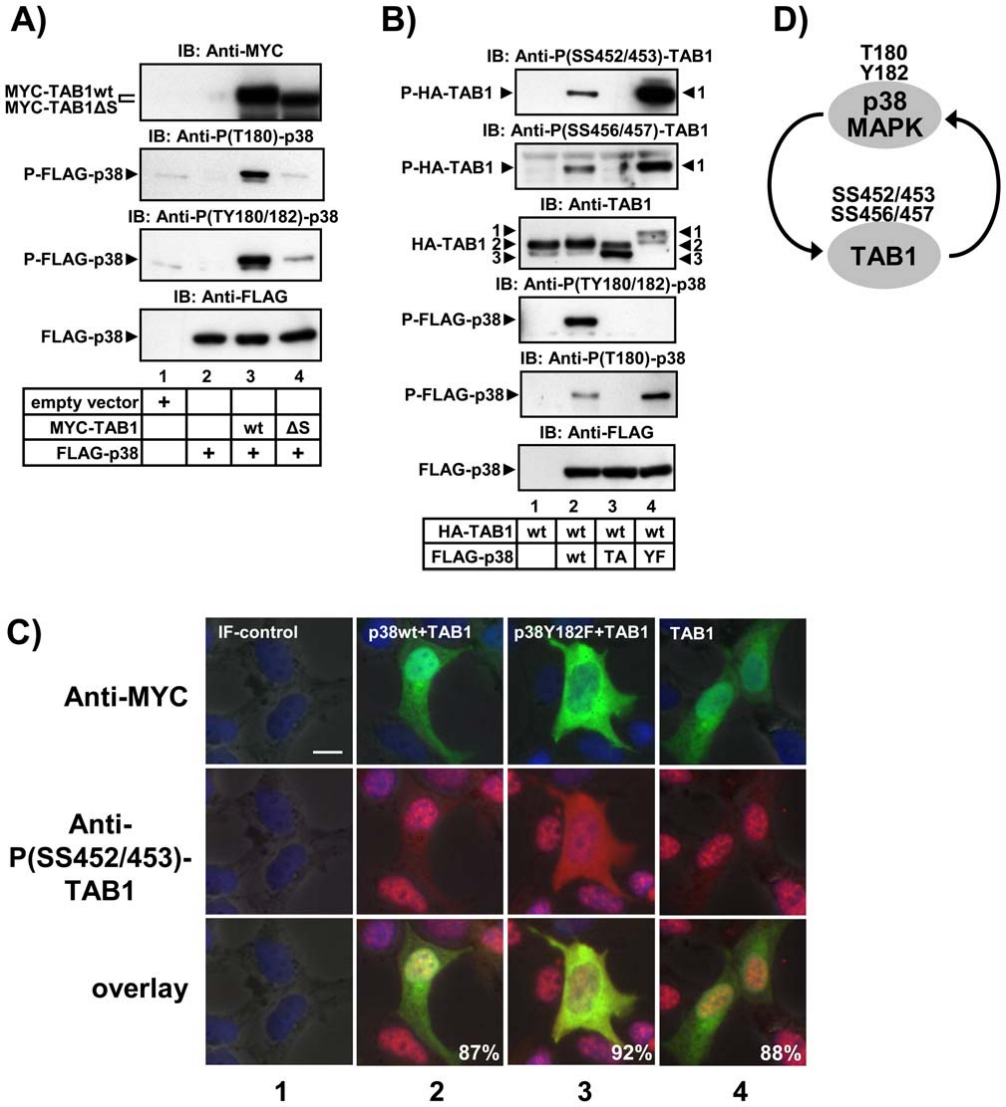
Amino acids 452–457 of TAB1 promote p38 MAPK phosphorylation and p38 promotes phosphorylation of TAB1. (A) HEK293IL-1R cells were transiently transfected with empty vector, or FLAG-p38 MAPK alone or in combination with MYC-TAB1 wild type (wt) or MYC-TAB1 lacking aa 452–457 (ΔS). After 48 h, aliquots of lysates were analyzed by IB for phosphorylation of p38 MAPK at T180/Y182 or at T180 and for expression of the transfected proteins using MYC or FLAG antibodies. (B) HEK293IL-1R cells were transiently transfected with expression vectors for HA-TAB1, FLAG-p38 MAPK wild type (wt) or versions with T180A (TA) or Y182F (YF) mutations as indicated. After 24 h aliquots of lysates were analyzed by IB for phosphorylation of TAB1 at SS452/453 and SS456/457, p38 MAPK at T180/Y182 or T180 and for expression of the transfected proteins using the indicated antibodies. C) HEK293IL-1R cells were transiently transfected with empty vector or expression vectors encoding MYC-TAB1 or FLAG-p38 MAPK wild type (wt) or the p38 MAPK Y182F (YF) mutation as indicated. After 24 h, cells were seeded for 24 h in μ-slides followed by double-immunofluorescence microscopy using anti MYC (green color) or anti phospho-TAB1 (red color) antibodies to detect TAB1 localization and phosphorylation at SS452/453. In IF-control, the primary antibodies were omitted. Nuclei were visualized by Hoechst staining (blue). Scale bar is 10 µM. Numbers indicate percentage of transfected cells with the respective phenotype. D) Scheme indicating mutual control of TAB1 and p38 MAPK involving aa 452–457.

In contrast to Ge *et al.* [24] we were unable to activate p38 MAPK with TAB1 *in vitro* precluding an analysis of aa 452–457 on the *in vitro* kinase activity of p38 MAPK (data not shown). Therefore, we tested if TAB1 was able to affect phosphorylation of p38 MAPK in intact cells. Both, TAB1 and p38 MAPK were co-expressed and the phosphorylation state of p38 MAPK was investigated by immunoblot analysis of whole cell lysates (Fig. 4A). In agreement with Ge *et al.* [24] we found that overexpression of TAB1 increased phosphorylation of p38 MAPK. In our experiments, this occurred primarily at T180 but also at Y182 in intact cells (Fig. 4A, lane 3). This effect was reduced by co-expressing TAB1ΔS, providing support for a TAB1-dependent activation mechanism of p38 MAPK that involves aa 452–457 (Fig. 4A, lane 4). We also tried to test the localization of TAB1 and p38 MAPK under conditions which induce strong TAB1 phosphorylation at SS452/453 or SS456/457. However, stress-related rounding up and detachment of cells under conditions of transfection of MKK62E or TAB1/TAK1, or, calyculin A or sorbitol treatment prevented microscopic analysis of TAB1 and p38 MAPK localization (data not shown) under the conditions established in the experiments shown in Fig. 1 and Fig. 3.

We, therefore, designed experiments to activate p38 MAPK by a separate strategy. Specifically, we used a p38 MAPK Y182F mutant which was shown by [38] to gain some kinase activity *in vitro* albeit to a much lower level than p38 MAPK fully phosphorylated at T180 and Y182 [38]. We applied this mutant of p38 MAPK to mimic modest activation of this pathway in the absence of any upstream trigger for analyzing p38 MAPK-specific effects on TAB1. Overexpression of wild type forms of p38 MAPK and TAB1 together resulted in increased phosphorylation of p38 MAPK as expected from the results shown in Fig. 4A. However, under this condition there was also increased phosphorylation of TAB1 at SS425/453 and SS456/457 (Fig. 4B, lane 2). The gain-of-function mutant p38 MAPK Y182F stimulated its own catalytic activity as assessed by increased autophosphorylation at T180 (Fig. 4B, lane 4). Moreover, when TAB1 was co-expressed with p38 MAPK Y182F, phosphorylation at both, SS452/453 and SS456/457 of TAB1 increased significantly, suggesting that active p38 MAPK is sufficient to phosphorylate these residues in intact cells (Fig. 4B, lane 4). Compared to co-expression of wild type p38 MAPK plus TAB1, co-expression of the active p38 MAPK Y182F mutant resulted in a primarily cytosolic localization of MYC-tagged TAB1 (Fig. 4C, columns 2 and 3). Moreover, p38 MAPK Y182F significantly increased TAB1 phosphorylation in the cytoplasm (Fig. 4C, column 3). This result suggests that phosphorylation of aa 452–457 of TAB1 is a means to promote cytosolic localization of TAB1. Collectively, the results shown in Fig. 4A to Fig. 4C suggest that aa 452–457 are engaged in TAB1-dependent activation of p38 MAPK but are also direct targets of p38 MAPK as schematically shown in Fig. 4D.

### Deletion of amino acids 452–457 and phospho-mimetic mutants of TAB1 target TAB1 and p38 MAPK to the cytoplasm

**Figure 5:**
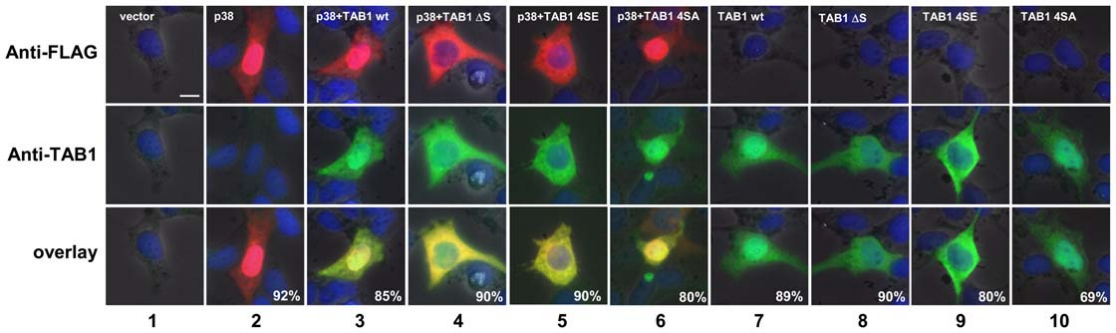
Mutations of aa 452–457 direct p38 MAPK and TAB1 to the cytoplasm. HEK293IL-1R cells were transiently transfected with empty vector or expression vectors for FLAG-p38 MAPK alone or in combination with MYC-TAB1 wild type (wt), MYC-TAB1 lacking aa 452–457 (ΔS), MYC-TAB1 SSSS452/453/456/457EEEE (MYC-TAB1 4SE), or MYC-TAB1 4SA. After 24 h, cells were seeded for 24 h in μ-slides followed by double-immunofluorescence microscopy using anti FLAG and anti TAB1 (H-300) antibodies to detect intracellular localization of p38 MAPK (1^st^ row, red) or TAB1 (2^nd^ row, green) by single or merged images (3^rd^ row). In IF-control, the primary antibodies were omitted. Nuclei were visualized by Hoechst staining (blue). Scale bar is 10 µM. Numbers indicate percentage of transfected cells with the respective phenotype.

To further reveal the role of aa 452–457 in p38 MAPK signaling, we analyzed the effects of deletion (TAB1ΔS), and phosphomimetic (TAB1 4SE) or phosphorylation-deficient (TAB1 4SA) mutants on both, p38 MAPK and TAB1 localization. In agreement with [39] we found in HEK293IL-1R cells that the majority of FLAG-p38 MAPK is localized in the nucleus of transfected cells (Fig. 5B, 2^nd^ column). This effect is not changed by co-expressed wild type MYC-TAB1 (Fig. 5B, 3^rd^ column). However, co-expression of TAB1ΔS or TAB1 4SE but not of a TAB1 4SA mutant resulted in an almost complete cytosolic re-localization of both, p38 MAPK and TAB1 (Fig. 4B, 4^th^ to 6^th^ column). These results suggested that the serine cluster or its phosphorylation regulate cytosolic retention of TAB1 and of p38 MAPK.

Collectively, the results shown in Fig. 4 and 5 indicate that p38 and TAB1 mutually control their subcellular localization and imply that aa 452–457 are involved in interactions of phospho-TAB1 and active p38 MAPK to control the cytoplasmic pool of both proteins.

### Amino acids 452–457 of TAB1 play a role in post-transcriptional gene regulation

**Figure 6:**
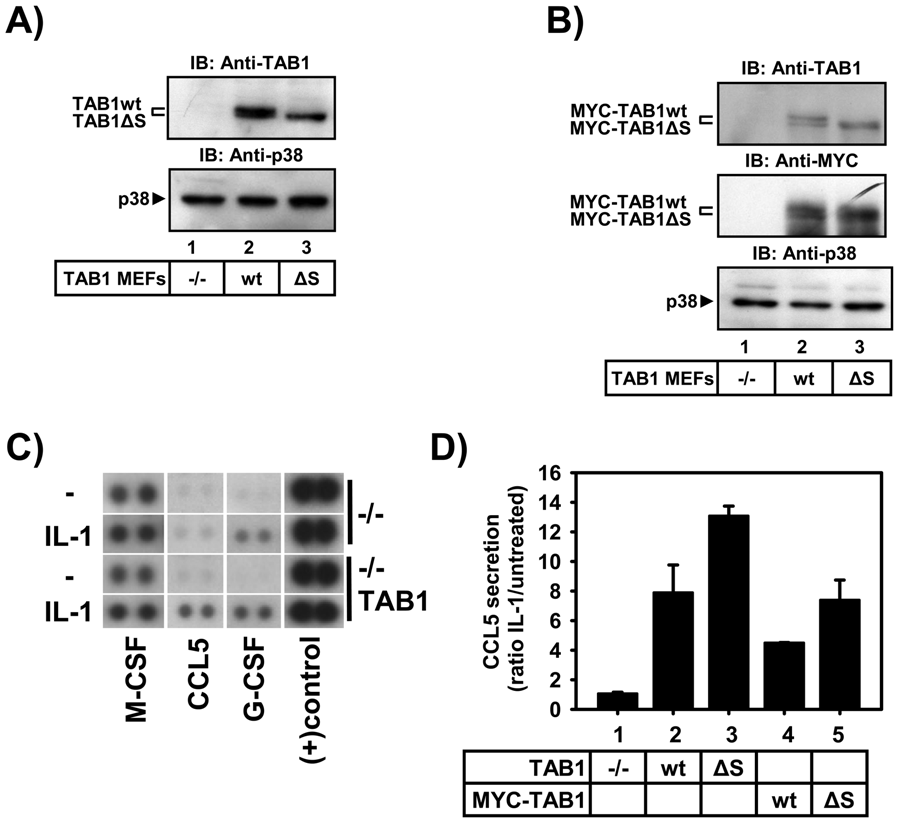
Amino acids 452–457 participate in modulation of TAB1-dependent CCL5 chemokine secretion in the IL-1 pathway. Murine embryonic fibroblasts were stably infected with retroviruses encoding untagged (A) or MYC-tagged (B) TAB1 wild type (wt) or TAB1 lacking aa 452–547 (ΔS). Lysates from pools of cells were analyzed for expression of the TAB1 and p38 MAPK proteins using the indicated antibodies. C) Cells from (A) were stimulated for 24 h with IL-1α (10 ng/ml) or were left untreated. Supernatants were analyzed for secretion of cytokines by antibody arrays. Results show images of raw data obtained for two IL-1-regulated proteins (CCL5, G-CSF), one unregulated but secreted protein (M-CSF) and a positive control. D) Supernatants of cells from (A) or (B) were treated as in C) and analyzed for CCL5 secretion by ELISA. Data were normalized for protein content of cell pellets harvested in parallel. Shown are the mean ratios +/− S.E.M. of IL-1-regulated CCL5 secretion from two independent experiments.

In order to assign additional biological functions to aa 452–457 of TAB1 we used stably reconstituted TAB1-deficient Mefs infected with retroviral expression vectors containing either untagged (Fig. 6A) or MYC-tagged (Fig. 6B) versions of TAB1 or TAB1ΔS. In line with previous studies [30], [33], [34], [40] we did not observe significant changes of IL-1-induced NF-κB, JNK and p38 MAPK pathways in TAB1-deficient Mefs (data not shown). As the data presented in Fig. 4 and Fig. 5 pointed to a cytosolic function of aa 452–457 of TAB1, and TAB1 siRNA were found to effect secretion of IL-8, IL-6, MCP-1 and GM-CSF in IL-1-treated HeLa cells [40] we screened the supernatants of TAB1-deficient and of reconstituted cells for secreted cytokines by antibody arrays. As shown in Fig. 6C, IL-1-induced CCL5 secretion was impaired in the absence of TAB1 and was restored upon reconstitution. Other IL1-induced genes such as G-CSF were unaffected by ablation of TAB1 suggesting a specific role of TAB1 in the IL-1 response. Compared to wild type TAB1, the IL-1-induced secretion of CCL5 was enhanced by approximately 30% by reconstituting cells with either untagged, or MYC-tagged versions of TAB1ΔS as assessed by specific ELISA (Fig. 6D).

**Figure 7:**
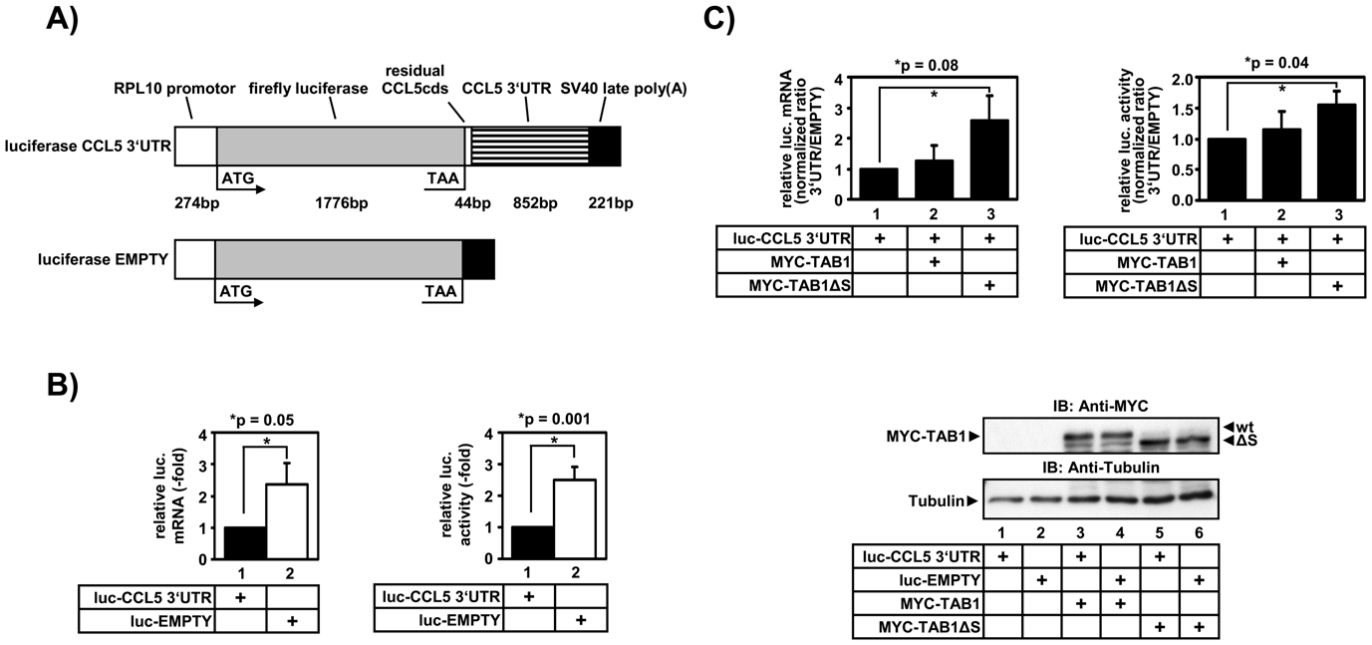
Amino acids 452–457 of TAB1 participate in mRNA-destabilization mediated by the *CCL5* 3′ untranslated region. A) Scheme of the luciferase encoding empty control vector and the construct harboring the *CCL5* 3′ UTR. B) A549 cells were transiently transfected with the control vector or the version carrying the *CCL5* 3′UTR together with SV-40-ß-gal. After 24 h, cells were splitted into two halves, one of which was used to extract total RNA and the other was lysed for determination of luciferase activity. Luciferase mRNA was measured by RT-qPCR and luciferase activity was assessed using a chemiluminescence assay and normalized for ß-galactosidase activity. Depicted is the relative mRNA expression and luciferase activity in the presence or absence of the *CCL5* 3′UTR. Data are shown as mean values +/− s.e.m. which were determined in 13 independent transfection experiments. C) Similar experiments as in B) were performed including co-expression of MYC-TAB1 wild type or MYC-TAB1ΔS. Shown is the mean ratio +/− s.e.m. of luciferase mRNA expression or activity as determined in at least seven independent transfection experiments. An increased ratio value indicates a reduced destabilizing effect of the *CCL5* 3′UTR relative to empty vector control. Lysates from transfected cells were analyzed for expression of TAB1 proteins using the indicated antibodies. Equal loading of lanes was confirmed using anti tubulin antibodies.

Further experiments were performed to assess at which step of gene regulation TAB1ΔS affected *CCL5* expression. As TAB1-deficient Mefs were difficult to transfect transiently we analyzed A549 cells which strongly upregulate CCL5 protein and mRNA in response to IL-1 in a TAK1-dependet manner (Fig. S4). A previously described *CCL5* promoter reporter gene construct [41] did not reveal any regulation by IL-1 and showed an inhibition of basal promoter activity upon co-expression of TAB1 wild type or TAB1ΔS (data not shown). We, therefore, fused the entire 3′ untranslated region (UTR) of the *CCL5* mRNA to a luciferase cDNA (Fig. 7A) to investigate post-transcriptional regulation of *CCL5*. Fusion of the *CCL5* 3′ UTR decreased luciferase mRNA and activity by 2–3-fold in reporter gene assays suggesting that the *CCL5* 3′ UTR conferred mRNA destabilization to the otherwise stable luciferase mRNA (Fig. 7B). Co-transfection of TAB1 did not alter this destabilizing effect, whereas co-transfection of TAB1ΔS increased steady state levels of the mRNA by 2.5-fold and luciferase activity by about 1.5-fold in seven independent transfection experiments (Fig. 7C). Together with the mainly cytosolic localization of the TAB1ΔS and TAB1 4SE mutants as shown in Fig. 5 these results suggest that aa 452–457 play a role in post-transcriptional control of cytokine-responsive mRNAs such *CCL5*.

## Discussion

TAB1 was the first TAK1-binding protein to be discovered [7]. TAB1 has also been implicated in an alternative allosteric activation mechanism of p38 MAPK [24]. Moreover, the protein is modified at multiple residues [20], [26]. Despite these findings, as outlined above the biological role of TAB1 in the TAK1-MKK3/6-p38 MAPK pathways or in any other biological process is not well understood.

Here, we add more facets to this apparent complexity of TAB1 regulation by identifying the serine cluster surrounding aa 452–457 as new phosphorylation sites and as a new functional domain within TAB1. These sites were detected by conventional biochemical approaches and were confirmed using phospho-specific antibodies. Importantly, none of these sites were discovered by previous mass spectrometry approaches [20], [42], [43] underscoring that a combination of different techniques is required to reveal all possible modifications in TAB1.

TAK1 and p38 MAPK phosphorylated TAB1 at SS452/453 and SS456/457 *in vitro* and in intact cells. Physiological phosphorylation at these sites was vey difficult to detect, but the available evidence suggests that a broader range of known TAK1- and p38-activating stimuli including cytokines and translational stressors targets these sites. Moreover, the strongest inducing condition was either overexpressing active TAK1 or p38 MAPK or blocking serine/threonine phosphatases by calyculin A implying that SS452/453 and SS456/457 are tightly controlled by reversible phosphorylation events. As we did not find a significant contribution of aa 452–457 to activation of TAK1, we focused on a putative role in p38 MAPK functions.

Experiments in transfected cells suggest that aa 452–457 played a role in cytosolic retention of both p38 MAPK and TAB1. Overexpressed p38 MAPK and its phosphorylated form is known to reside in the nucleus [39]. However, p38 MAPK localization is highly dynamic, as p38 MAPK redistributes to the cytoplasm upon stress via the nuclear export signal of its substrate and interaction partner MK2 [39], [44]. Both studies showed that the trafficking of p38 MAPK is determined by the stoichiometry of p38 MAPK:MK2 complexes [39], [44]. Another study showed in rat cardiomyocytes that p38α MAPK travels not only with MK2 but also together with TAB1. Thus, TAB1 versions fused to either a cytosolically located red fluorescent protein (RFP-TAB1) or to a nuclear localization signal (NLS-TAB1) directed a GFP-p38 MAPK fusion protein towards the cytoplasm or the nucleus, respectively [37]. Our data strengthening this concept and suggest that the serine cluster of aa 452–457 is involved in nuclear targeting, as its deletion promotes a primarily cytosolic localization of TAB1 and of p38 MAPK (Fig. 5). Interestingly, the phosphomimetic mutant TAB1 4SE has the same phenotype, whereas the TAB1 4SA mutant promotes nuclear localization suggesting that in the natural TAB1 protein phosphorylation of the serine rich cluster targets TAB1 and p38 MAPK to the cytoplasm. A mechanistic explanation might be that TAB1 through aa 452–457 binds to an unknown nuclear partner protein. This interaction is relieved upon phosphorylation at aa 452–457. Released TAB1 then redistributes to the cytoplasm carrying p38 MAPK with it. In this model, lack of aa 452–457 would impair binding, whereas prevention of phosphorylation as shown by the TAB1 4SA mutant would stabilize binding to the speculative nuclear partner protein of TAB1, explaining the cytoplasmic versus nuclear localization of TAB1 ΔS and TAB1 4SA as shown in Fig. 5, respectively.

Only three partial 3-D structures of TAB1 are available, derived from crystals of either N-terminal fragments of aa 7–402 [25] or aa 1–370 [45] or from a crystal structure of a fusion protein of aa 468–504 fused to the TAK1 catalytic domain (aa 31–303) [46]. The serine cluster described in our study is not part of these structures and is also not involved in p38 MAPK binding [22]. It does neither resemble a classical basic nuclear localization signal (NLS) [47] nor a nuclear translocation signal (NTS) [48]. Hence, it apparently represents a novel regulatory domain in TAB1 which participates in fine-tuning of the p38 MAPK signaling pathway by contributing to p38 MAPK localization and autoactivation of the kinase independent from the classical MAP3K-MAP2K-MAP kinase cascades.

We also tried to validate a role of aa 452–457 in regulating p38 MAPK:TAB1 interactions by co-immunoprecipitation. However, there was little if any stable interaction of p38 MAPK with TAB1 in these assays (data not shown). This would suggest that the protein:protein interaction is either very labile or indirect. However, TAB1 promoted p38 MAPK phosphorylation primarily at T180 and the gain-of-function mutant of p38 MAPK Y182F promoted TAB1 phosphorylation at aa 452–457 adding further evidence for a mutual control of both proteins (Fig. 4).

The results presented in this study can be reconciled by the following model: in unstimulated cells, a low level of p38 MAPK activity is observed that is caused by a TAB1-dependent activation mechanism involving p38 MAPK auto-phosphorylation at T180. Conversely, TAB1 can be phosphorylated by p38 MAPK at serines 452–457 (Fig. 1, 2, 4) retaining the phosphorylated protein primarily in the cytoplasm (Fig. 4). Normally, most TAB1 in cells is kept unphosphorylated by serine/threonine phosphatases and is found in the nucleus. Accordingly, we identified three conditions that shift this balance towards strong phosphorylation at aa 452–457 of TAB1: (i) blockade of phosphatases by calyculin A (Fig. 3), (ii) strong osmotic or translational stress (Fig. 3) and (iii) ectopic expression of TAK1, p38 MAPK plus MKK62E or the gain-of-function mutant p38 MAPK Y182F together with TAB1 (Fig. 1, 2, 4). Both, TAB1 versions lacking aa 452–457 or carrying phospho-mimetic mutations trapped p38 MAPK in the cytoplasm (Fig. 5). This suggests that phosphorylated TAB1 may support post-transcriptional functions of p38 MAPK such as regulation of *CCL5* mRNA stability (Fig. 7) and secretion (Fig. 6).

Although TAK1 is another kinase that phosphorylates aa 452–457, we did not find any general effects of TAB1 mutants on overall TAK1 activation in the settings analyzed in this study. Therefore, TAB1 molecules phosphorylated at aa 452–457 by TAK1 may have unknown roles in stimulus-specific activation of TAK1 pathways.

In summary, our study suggests an intimate mutual control of p38 MAPK and its allosteric activator TAB1. Both proteins control dynamically each other's phosphorylation status, activity and subcellular localization. Our results further establish TAB1 as a multi-site phosphorylated protein and provide another piece in the puzzle of solving the function of the “enigmatic” TAB1.

In depth clarification of the underlying mechanisms will require much more extensive studies for identifying TAB1-interacting molecules that are controlled by phosphorylation of aa 452–457 and that are involved in TAB1-dependent biological functions.

## Materials and Methods

### Cells and materials

HEK293 cells stably expressing the IL-1 receptor (HEK293IL-1R) [15], TAB1 −/− murine embryonic fibroblast lines, a kind gift of Kunihiro Matsumoto [33], and A549 lung epithelial carcinoma cells (catalogue number ACC 107, obtained from DSMZ, German Collection of Microorganisms and Cell Cultures) were cultured in Dulbecco's modified Eagle's medium (DMEM), complemented with 10% fetal calf serum, 2 mM L-glutamine, 100 U/ml penicillin, 100 µg/ml streptomycin. Antibodies against the following proteins or peptides were used: actin (JLA20; EMD) and TAK1 (M-579, sc-7162), TAB1 (H-300, sc-13956), GST (Z-5, sc-459), tubulin (TU-02, sc-8035), TAB2 (K-20, sc-11851), rabbit IgG (sc-2027) all from Santa Cruz, MYC (9E10), HA (12CA5), GFP (clone 7.1 and 13.1) all from Roche, FLAG M2 (F1804, Sigma), P(T180/Y182)-p38 MAPK (36–850, Invitrogen), P(T180)-p38 MAPK (02504), P(S438)-TAB1 (09519) both from Acris, TAK1 (4505), P(T187)-TAK1 (4536), TAB1 (C25E9) all from Cell signaling. p38 MAPK [49] and P(T187)-TAK1 [6]. Purified polyclonal antibodies that recognize specific phosphorylation sites in human TAB1 were raised by Eurogentec against the following peptides: TNTHTQpSpSSSSSD (SS452/3) and TQSSSSpSpSDGGLF (SS456/7), where pS denotes a phosphorylated serine.

### Plasmids, transfections, reporter gene assays, RT-qPCR

The expression vectors pCMV-FLAG-p38 MAPK, pCDNA3-MKK62E [50], [51], pCMV-HA-TAK1, pCMV-HA-TAK1K63W, pUHC13-3-IL-8 promotor, pSV40-ß-Galactosidase [11], pCDNA3-GFP-YopP, pCDNA3-GFP-TAK1, pCS2MT-MYC-TAB1, pCDNA3-HA-TAB1, pCDNA3-HA-TAB1 S438A, pCDNA3-HA-TAB1 S423/T431AA (STS/AAS), pCDNA3-HA-TAB1 S423/T431/S438AAA (STS/AAA), pDEST26-HIS-Ubiquitin [15] and pGEX-GST-TAB1 [17] have been described. The following plasmids were constructed by standard cloning techniques: pCDNA3-HA-TAB1 lacking aa 452–457 (ΔS), pCDNA3-HA-TAB1 S438A ΔS, pCDNA3-HA-TAB1 S425A, pCDNA3-HA-TAB1 S452A, pCDNA3-HA-TAB1 S453A, pCDNA3-HA-TAB1 S456A, pCDNA3-HA-TAB1 S457A, pCDNA3-HA-TAB1 S452E, pCDNA3-HA-TAB1 S453E, pCDNA3-HA-TAB1 S456E, pCDNA3-HA-TAB1 S457E, pCS2MT-MYC-TAB1 ΔS, pCS2MT-MYC-TAB1 S452/3/6/7A (4SA), pCS2MT-MYC-TAB1 S452/3/6/7E (4SE), pGEX-GST-TAB1 4SA, pCMV-FLAG-p38 T180A (TA), pCMV-FLAG-p38 Y182F (YF), pCMV-FLAG-p38 T180/Y182AF (AF). Mutations were performed using the Stratagene site directed mutagenesis kit, a modification of which [52] was also used for deletion of the region encoding aa 452–457 in TAB1. pSGG-luciferase- *CCL5* 3′UTR and pSGG-luciferase-EMPTY (Fig. 7A) were purchased from switch gear genomics.

For retroviral expression, a cDNA encoding TAB1 was amplified from pCDNA3.1-HA-TAB1 [15] using the primers (se: 5′-GCGCGAATTCAATGGCGGCGCAGAGG-3′; as:5′-GCGCCTCGAGTATCGATACTACGCTGCTGTCACCACG-3′), cloned into pCR-Blunt II-TOPO and then subcloned into the EcoRI and XhoI sites of pCS2-MT. A ClaI fragment of pCS2-MT-MYC_(6)_-TAB1 was then cloned into the ClaI site of pM5XNeo to generate pM5XNeo-MYC-TAB1. pM5XNeo-TAB1 was generated by excising the MYC-tags by EcoRI digestion and religation. Recombinant retroviruses and infections of Mef cells were done exactly as described in [49]. Stable cell lines were selected using 1 mg/ml G418.

Calcium phosphate transfections and reporter gene assays were performed as described [53], [54].

Lipofectamine transfections of A549 cells were performed according to the manufacturer's instructions (Invitrogen, Lipofectamine LTX and PLUS reagents Cat. no. 15338). Specifically, A549 cells were seeded at 5–6×10^5^ in 9,4 mm wells. 24 h later cells were transfected in duplicates with 2.5 µg plasmid DNA containing 0.4 µg pSV40-ß-galactosidase + 0.1 µg pSGG-luciferase- *CCL5* 3′UTR or pSGG-luciferase-EMPTY in combination with 2 µg empty vector control or pCS2MT-MYC-TAB1wt or pCS2MT-MYC-TAB1ΔS) and 3 µl PLUS reagent and 3.75 µl lipofectamine in DMEM per well. After 6 h at 37°C, 5% CO_2_, the medium was complemented with 10% fetal calf serum, 2 mM L-glutamine, 100 U/ml penicillin, 100 µg/ml streptomycin. The next day, cells were harvested and splitted into two halves one of which was used to extract mRNA and the other was lysed for determination of luciferase activity or equal expression of the TAB1 constructs.

RT-qPCR was performed as described [54]. cDNAs were amplified using assays on demand (Applied Biosystems) for *CCL5* (Hs00171085_m1), ß-actin (Hs99999903_m1) and luciferase (se-primer: 5′-GCGCAGCTTGCAAGACTATAAG-3′, as-primer: 5′-TTGTCGATGAGAGTGCTCTTAGC-3′, probe: 5′-CTGGTGCCCACACTAT-3′).

### Cell lysis and immunoprecipitations

Unless stated otherwise, cells were lysed in cell lysis buffer (50 mM TrisHCl, pH 7.5, 100 mM NaCl, 0,1 mM EGTA, 1 mM EDTA, 1% Triton X-100, 50 mM NaF, 1 µM Microcystin, 1 mM Na_3_VO_4_, 5 mM sodium pyrophosphate, 0,1% ß-mercaptoethanol and a Roche protease inhibitor mix). HEK293IL-1R cells (Fig. S3B) and A549 cells (Fig. 7) transfected for reporter gene assays were lysed in ß-galactosidase lysis buffer as described [53].

For immunoprecipitation of p38 MAPK (Fig. 2A, 2B), cells were lysed in (10 mM Tris, pH 7.05, 30 mM NaPPi, 50 mM NaCl, 1% Triton X-100, 2 mM Na_3_VO_4_, 50 mM NaF, 20 mM ß-glycerophosphate, 1 mM PMSF, 1 µg/ml leupeptin, 1 µg/ml pepstatin, and 1 µM microcystin). p38 MAPK was immunoprecipitated from 1.5 mg cell extract protein in (1×Tris-buffered saline (TBS), 1% Triton X-100, 1 mM Na_3_VO_4_, 2 mM DTT, 50 mM NaF) using rabbit polyclonal anti p38 MAPK antibodies [49] (Fig. 2A) or FLAG antibodies (Fig. 2B) coupled to protein-G-Sepharose 4 Fast Flow (GE Healthcare). Beads were washed twice in the same buffer, once in washing buffer containing (50 mM Tris, pH 7.5, 10 mM MgAc, 0.1% ß-mercaptoethanol) and redissolved in 10 µl of washing buffer for *in vitro* kinase assays.

For immunoprecipitation of TAK1-TAB1 (Fig. 2C) or GFP-TAK1 (Fig. S3A), cell extracts were incubated with protein G Sepharose 4 Fast Flow coupled to 1 µg of MYC (9E10) or to 1 µg of GFP (clone 7.1 and 13.1) antibodies for 2–6 h, respectively, with gentle rocking at 4°C. Beads were then washed two times with cell lysis buffer plus 0,5 M NaCl and once with washing buffer. Beads were redissolved in 10 µl washing buffer for *in vitro* kinase assay or were splitted into two halves one of which was boiled for 5 min in 2× Roti-Load (Roth) before loading on 8% SDS-PAGE. The other half was redissolved in 10 µl washing buffer for *in vitro* kinase assay.

For immunoprecipitation of endogenous TAB1 (Fig. 3B) cells were lysed in (50 mM HEPES, pH 7.4, 50 mM NaCl, 1% Tween 20, 2,5 mM EGTA, 1 mM EDTA, 1 mM NaF,10 mM ß-glycerophosphate, 0,1 mM Na_3_VO_4_, 1 mM PMSF, Roche protease inhibitor mix, 1 mM DTT, 1 µM microcystin), sonified 3×20 seconds on ice and centrifuged at 100.000×g for 20 min at 4°C. 1.5 mg of the supernatant was used for IP with 1.5 µg of anti TAB1 (H-300) or IgG (sc-2027) antibodies coupled for 1 h to 15 µl of Trueblot anti-mouse Ig immunoprecipitation beads (e-bioscience, 00-8811-25). After 2 h, beads were washed three times in the lysis buffer including 450 mM NaCl. Proteins were eluted by boiling in 2× Roti-Load before loading on SDS-PAGE.

Immunoprecipitation of the endogenous TAK1-TAB1-TAB2 complex (Fig. S3C) using TAB2 (K-20, Santa Cruz) antibodies followed by *in vitro* kinase assay to measure TAK1 kinase activity using recombinant HIS_(6)_-MKK6 and γ[^32^P]ATP as substrates was performed as described [15].

### *In vitro* kinase assays

Unless stated otherwise, *in vitro* kinase assays were carried out at 37°C for 30 min with agitation in 30 µl of reaction buffer containing 10 µl H_2_O including approximately 1 µg of recombinant GST or HIS-tagged protein, 10 µl of redissolved protein G Sepharose beads containing kinases immunoprecipitated from cell extracts and 10 µl (100 mM Tris pH 7.5, 20 mM MgAc, 0.4–2 mM ATP, 0,2% ß-mercaptoethanol). For the experiments shown in Fig. 2A, C, reaction mixtures were spun down and the supernatants (∼30 µl) were incubated with glutathione Sepharose (GE Healthcare) slurry (equilibrated in cell lysis buffer) at 37°C for 30 min with agitation for purification of the recombinant GST-tagged protein. Adsorbed GST-fusion proteins were washed three times with lysis buffer. Finally, beads were boiled for 5 min in 2× Roti-Load and proteins separated by SDS-PAGE and detected by western blotting. In the experiment shown in Fig. S3A, 5 µCi γ[^32^P]ATP were added to the reaction buffer and phosphorylated proteins were separated by SDS-PAGE and visualized by autoradiography.

### Immunostaining and fluorescence microscopy

HEK293IL-1R cells were cultured in 9,4 mm wells. After 24 h of transfection, cells were seeded for 24 h in μ-slides VI (Ibidi). After washing, cells were fixed with 4% paraformaldehyde in Hank's BSS (PAA Laboratories) for 5 min, blocked with 10% normal donkey serum (Dianova) for 30 min and incubated with primary and secondary antibodies diluted in Hank's BSS containing 0.1% Saponin (Sigma) for 2 h at room temperature. The primary antibodies were anti-TAB1 (H-300, Santa Cruz, 1∶100), anti-FLAG M2 (F1804, Sigma, 1∶100), anti-MYC 9E10 (Roche, 1∶200) and anti-P(SS452/3)-TAB1 (1∶100). Cy3-congugated (Chemicon, 1∶200) and FITC-conjugated (Sigma, 1∶100) secondary antibodies were used. For controls, primary antibodies were omitted. Nuclei were stained with Hoechst 33342 (Invitrogen). Fluorescence imaging was performed on a Leica DM IRE2 fluorescence microscope. Captured images were analysed with the Leica FW4000 Fluorescence Workstation software.

### Immunoblotting and ELISA

Immunoblotting was performed essentially as described [54]. Proteins were separated on 7.5–10% SDS-PAGE and electrophoretically transferred to PVDF membranes (Millipore). After blocking with 1% or 5% dried milk in Tris-HCl-buffered saline/0.05% Tween (TBST) for 1 h, membranes were incubated for 12–24 h with primary antibodies, washed in TBST and incubated for 1–2 h with the peroxidase-coupled secondary antibody. Proteins were detected by using enhanced chemiluminescence (ECL) systems from Pierce, Millipore or GE Healthcare.

### Cytokine arrays and ELISA

The mouse cytokine array panel A kit (ARY006) and the mouse CCL5 duoset (DY478) were used according to the manufacturer's instructions (R&D Systems, Inc.).

### Ubiquitination assays

Ubiquitination of TAB1 and TAK1 was determined as described in [15] with the following modifications. Briefly, one 75 cm^2^ flask of cells were transfected with 10 µg of expression vectors for HA-TAK1, MYC-TAB1, p38 MAPK plus MKK62E (5 µg each) and 20 µg of pDest26-HIS_(6)_-Ubiquitin or pDest26-HIS_(6)_. Total amount of DNA was adjusted to 50 µg by adding pCDNA3.1. After 24 h cells were lysed in 1 ml of lysis buffer (6 M guanidine-HCl, 0,1 M Na_2_HPO_4_/NaH_2_PO_4_, 10 mM imidazole, pH 8.0). Lysates were sonicated to shear DNA and cleared by centrifugation for 5 min at 15.000×g. 3 mg of lysate protein were incubated with 50 µl of Ni^2+^- NTA agarose for 3 h at room temperature. Beads were collected by centrifugation, washed twice in lysis buffer, twice in a buffer containing 1 volumes of lysis buffer and three volumes of buffer TI (25 mM Tris pH 6.8, 20 mM imidazole, adjusted to pH 6,8), and twice with buffer TI alone. Bound proteins were eluted for 5 min at 95°C in SDS PAGE sample buffer. 120 µg of proteins from the initial lysate (input) were precipitated by adding one volume of icecold TCA (10%). The pellet was collected at 15.000×g for 15 min at 4°C, washed once in two volumes of icecold EtOH (100%), dried in a speed vac and resuspended in 100 µl of SDS PAGE sample buffer at 95°C for 5 min. Bound and input protein samples were separated by 8%SDS-PAGE including 4.5% glycerol and analysed by western blotting.

## Supporting Information

Figure S1 **Evidence for new phosphorylation sites in TAB1 in addition to S423, T431 and S438.** A) HEK293IL-1R cells were transiently transfected with expression vectors for HA-TAB1 wild type, or versions in which S423/T431/S438 (STS) were mutated to alanine as indicated alone or in combination with GFP-YopP, FLAG-p38 MAPK plus MKK62E or HA-TAK1. B) A similar experiment as in A) was performed using a TAB1 mutant in which S425 was mutated to alanine (HA-TAB1 S425A). 24 h later, cells were lysed followed by immunoblotting (IB) to detect HA-TAK1, FLAG-p38 MAPK or HA-TAB1 using the indicated antibodies. Black arrowheads indicate the three forms of TAB1 (numbered 1–3) with different mobility on SDS-PAGE as previously described by us [15]. Explanation: As shown in Fig. S1A, a retarded mobility form 1 of TAB1 is induced by co-expression of MKK6-p38 MAPK (Fig. S1A, lane 3) or by TAB1-activated TAK1 (Fig. S1A, lane 4). In the absence of stimulation, form 2 shows intermediate mobility and represents a constitutive form of TAB1 which is found in unstimulated cells (Fig. S1A, lane 1). Form 3 of TAB1 displays fastest mobility and is observed by either mutating S438 (Fig. S1A, lane 13) or by intracellular co-expression of the bacterial protease YopP (Fig. S1A, lane 2) which we have shown previously to inhibit the TAK1-MKK6-p38 MAPK pathway [15]. In line with this result, TAB1 form 3 is also found in cells lacking p38α MAPK or treated with SB203580 [15], implying that S438 is constitutively phosphorylated by a low level of active p38 MAPK found in unstimulated cells. However, a version of TAB1 in which S423, T431 and S438 were mutated to alanine was still shifted by MKK6-activated p38 MAPK (Fig. S1A, lane 7) or by TAB1-activated TAK1 (Fig. S1A, lane 8) suggesting that active p38 MAPK and TAK1 phosphorylate novel sites in TAB1 in addition to the three well-characterized S423, T431 and S438 residues.
(TIF)

Figure S2 **Validation of phospho-specific antibodies recognizing phosphorylation of SS452/453 and SS456/457 of TAB1.** HEK293IL-1R cells were transiently transfected with expression vectors for FLAG-p38 MAPK plus MKK62E (A) or HA-TAK1 (B, C) in the indicated combinations with wild type HA-TAB1 (wt) or versions in which serines 452, 453, 456, 457 were point mutated to alanine (A, B) or to glutamic acid (C). 24 h later, cells were lysed and expression of FLAG-p38 MAPK or HA-TAK1 and expression or phosphorylation of HA-TAB1 was analyzed by immunoblotting (IB) using the indicated antibodies. Black arrowheads indicate forms of TAB1 with different mobility as described in the legend of Fig. 1. D) Scheme summarizing the differential effects of MKK62E-p38 MAPK or TAK1-TAB1 on phosphorylation of individual serines in cluster of aa 452–457. Explanation: As derived from individual S-A mutations, the antibodies reacted with all phosphorylated serines and each residue could be phosphorylated by activated p38 MAPK (Fig. S2A). Likewise, overexpression of TAK1 and TAB1 caused phosphorylation of SS452/453 and SS456/457 (Fig. S2B, lane 7). However, in contrast to stimulation of phosphorylations by active p38 MAPK, mutation of S453 to alanine prevented phosphorylation at S456/457 (Fig. S2B, lane 9), while mutation of S456 to alanine significantly weakened phosphorylation at SS452/453 (Fig. S2C, lane 10). Replacing S453 or S456 by phosphomimetic glutamic acid residues restored phosphorylation of neighbouring S456 or S453 residues, respectively, in response to TAK1 (Fig. S2C, lanes 8, 9). Hence, there is a mutual influence of phosphorylations within the serine cluster of TAB1 which is only seen when TAB1 is in complex with TAK1 but not with activated p38 MAPK, although both kinases can phosphorylate all four residues in intact cells (summarized in Fig. 2D).
(TIF)

Figure S3 **Amino acids 452–457 play only a marginal role in TAK1 activation and in ubiquitination of the TAK1-TAB1 complex.** A) HEK293IL-1R cells were transiently transfected with GFP-TAK1 and the indicated TAB1 expression vectors. 24 h later, cells were lysed, TAK1 was immunoprecipitated using GFP antibodies and its kinase activity was determined *in vitro* using recombinant HIS_(6)_-MKK6 and ^32^P-ATP as substrates. Reaction mixtures were separated on SDS-PAGE and phosphorylated MKK6 was detected by autoradiography (left panel) as described previously [15]. TAK1 and TAB1 proteins and phosphorylated TAK1 contained in the immunoprecipitates were validated using the indicated antibodies (right panel). B) HA-TAK1 and the indicated MYC-TAB1 versions were co-transfected with IL-8 promoter luc. constructs. 24 h later, cells were lysed and luciferase activity was determined. The graph shows the mean +/− s.e.m. of the relative IL-8 promoter activity from 5 independent transfections. As shown in the lower panel for one representative experiment, lysates were analyzed in parallel for equal expression of transfected proteins. C) TAB1-deficient Mefs were stably infected with retroviruses encoding wild type TAB1 or the indicated mutants. Cells were treated for 30 min with 50 nM calyculin A as indicated or were left untreated. Lanes 8 and 9 represent samples from wild type Mefs. The TAK1 complex was immunoprecipitated using TAB2 antibodies as described in [15] and basal TAK1 activity was assessed by radioactive *in vitro* kinase assays as described in A). Lane 1 indicates a sample in which the cell extract was omitted in the kinase reaction to control for autophosphorylation of bacterially expressed HIS_(6)_-MKK6. D) HEK293IL-1R cells were transiently transfected with empty vector or an ubiquitin expression vector (HIS-Ubiquitin) and combinations of epitope-tagged expression vectors for TAK1, TAB1, p38 MAPK and MKK62E as indicated. 24 h after transfection, cells were lysed in denaturing buffer. Ubiquitinated proteins were purified on Ni^2+^-NTA agarose. Lysates and affinity purified proteins were analyzed by immunoblotting using the indicated antibodies. Details are described in the methods section and in [15].
(TIF)

Figure S4 **TAK1-dependent activation of the** ***CCL5*** **gene in A549 cells.** A459 lung epithelial carcinoma cells were treated for 30 min with the TAK1 inhibitor 5Z-7-oxozeaenol (1 µM) followed by IL-1α (10 ng/ml) for 6 h, IL-1 alone or were left untreated. Thereafter, CCL5 secretion in the supernatant (A) or *CCL5* mRNA expression (B) were determined by specific ELISA and RT-qPCR, respectively. Shown are mean values +/− s.e.m. form 3 independent experiments.
(TIF)

## References

[1] Gaestel M, Kotlyarov A, Kracht M (2009). Targeting innate immunity protein kinase signalling in inflammation.. Nat Rev Drug Discov.

[2] Wan YY, Chi H, Xie M, Schneider MD, Flavell RA (2006). The kinase TAK1 integrates antigen and cytokine receptor signaling for T cell development, survival and function.. Nat Immunol.

[3] Sato S, Sanjo H, Takeda K, Ninomiya-Tsuji J, Yamamoto M (2005). Essential function for the kinase TAK1 in innate and adaptive immune responses.. Nat Immunol.

[4] Mizukami J, Takaesu G, Akatsuka H, Sakurai H, Ninomiya-Tsuji J (2002). Receptor activator of NF-kappaB ligand (RANKL) activates TAK1 mitogen-activated protein kinase kinase kinase through a signaling complex containing RANK, TAB2, and TRAF6.. Mol Cell Biol.

[5] Ninomiya-Tsuji J, Kishimoto K, Hiyama A, Inoue J, Cao Z (1999). The kinase TAK1 can activate the NIK-I kappaB as well as the MAP kinase cascade in the IL-1 signalling pathway.. Nature.

[6] Singhirunnusorn P, Suzuki S, Kawasaki N, Saiki I, Sakurai H (2005). Critical roles of threonine 187 phosphorylation in cellular stress-induced rapid and transient activation of transforming growth factor-beta-activated kinase 1 (TAK1) in a signaling complex containing TAK1-binding protein TAB1 and TAB2.. J Biol Chem.

[7] Shibuya H, Yamaguchi K, Shirakabe K, Tonegawa A, Gotoh Y (1996). TAB1: an activator of the TAK1 MAPKKK in TGF-beta signal transduction.. Science.

[8] Takaesu G, Kishida S, Hiyama A, Yamaguchi K, Shibuya H (2000). TAB2, a novel adaptor protein, mediates activation of TAK1 MAPKKK by linking TAK1 to TRAF6 in the IL-1 signal transduction pathway.. Mol Cell.

[9] Jin G, Klika A, Callahan M, Faga B, Danzig J (2004). Identification of a human NF-kappaB-activating protein, TAB3.. Proc Natl Acad Sci U S A.

[10] Ishitani T, Takaesu G, Ninomiya-Tsuji J, Shibuya H, Gaynor RB (2003). Role of the TAB2-related protein TAB3 in IL-1 and TNF signaling.. EMBO J.

[11] Holtmann H, Enninga J, Kalble S, Thiefes A, Dorrie A (2001). The MAPK kinase kinase TAK1 plays a central role in coupling the interleukin-1 receptor to both transcriptional and RNA-targeted mechanisms of gene regulation.. J Biol Chem.

[12] Cheung PC, Nebreda AR, Cohen P (2004). TAB3, a new binding partner of the protein kinase TAK1.. Biochem J.

[13] Wang C, Deng L, Hong M, Akkaraju GR, Inoue J (2001). TAK1 is a ubiquitin-dependent kinase of MKK and IKK.. Nature.

[14] Kanayama A, Seth RB, Sun L, Ea CK, Hong M (2004). TAB2 and TAB3 activate the NF-kappaB pathway through binding to polyubiquitin chains.. Mol Cell.

[15] Thiefes A, Wolf A, Doerrie A, Grassl GA, Matsumoto K (2006). The Yersinia enterocolitica effector YopP inhibits host cell signalling by inactivating the protein kinase TAK1 in the IL-1 signalling pathway.. EMBO Rep.

[16] Sakurai H, Nishi A, Sato N, Mizukami J, Miyoshi H (2002). TAK1-TAB1 fusion protein: a novel constitutively active mitogen-activated protein kinase kinase kinase that stimulates AP-1 and NF-kappaB signaling pathways.. Biochem Biophys Res Commun.

[17] Sakurai H, Miyoshi H, Mizukami J, Sugita T (2000). Phosphorylation-dependent activation of TAK1 mitogen-activated protein kinase kinase kinase by TAB1.. FEBS Lett.

[18] Kishimoto K, Matsumoto K, Ninomiya-Tsuji J (2000). TAK1 mitogen-activated protein kinase kinase kinase is activated by autophosphorylation within its activation loop.. J Biol Chem.

[19] Ono K, Ohtomo T, Sato S, Sugamata Y, Suzuki M (2001). An evolutionarily conserved motif in the TAB1 C-terminal region is necessary for interaction with and activation of TAK1 MAPKKK.. J Biol Chem.

[20] Cheung PC, Campbell DG, Nebreda AR, Cohen P (2003). Feedback control of the protein kinase TAK1 by SAPK2a/p38alpha.. EMBO J.

[21] Ge B, Xiong X, Jing Q, Mosley JL, Filose A (2003). TAB1beta (transforming growth factor-beta-activated protein kinase 1-binding protein 1beta ), a novel splicing variant of TAB1 that interacts with p38alpha but not TAK1.. J Biol Chem.

[22] Zhou H, Zheng M, Chen J, Xie C, Kolatkar AR (2006). Determinants that control the specific interactions between TAB1 and p38alpha.. Mol Cell Biol.

[23] Kang YJ, Seit-Nebi A, Davis RJ, Han J (2006). Multiple activation mechanisms of p38alpha mitogen-activated protein kinase.. J Biol Chem.

[24] Ge B, Gram H, Di Padova F, Huang B, New L (2002). MAPKK-independent activation of p38alpha mediated by TAB1-dependent autophosphorylation of p38alpha.. Science.

[25] Conner SH, Kular G, Peggie M, Shepherd S, Schuttelkopf AW (2006). TAK1-binding protein 1 is a pseudophosphatase.. Biochem J.

[26] Mendoza H, Campbell DG, Burness K, Hastie J, Ronkina N (2008). Roles for TAB1 in regulating the IL-1-dependent phosphorylation of the TAB3 regulatory subunit and activity of the TAK1 complex.. Biochem J.

[27] Hanada M, Ninomiya-Tsuji J, Komaki K, Ohnishi M, Katsura K (2001). Regulation of the TAK1 signaling pathway by protein phosphatase 2C.. J Biol Chem.

[28] Kajino T, Ren H, Iemura S, Natsume T, Stefansson B (2006). Protein phosphatase 6 down-regulates TAK1 kinase activation in the IL-1 signaling pathway.. J Biol Chem.

[29] Liu Q, Busby JC, Molkentin JD (2009). Interaction between TAK1-TAB1-TAB2 and RCAN1-calcineurin defines a signalling nodal control point.. Nat Cell Biol.

[30] Kettner-Buhrow D, Dittrich-Breiholz O, Schneider H, Wolter S, Resch K (2006). Small interfering RNAs generated by recombinant dicer induce inflammatory gene expression independent from the TAK1-NFkappaB-MAPK signaling pathways.. Biochem Biophys Res Commun.

[31] Takaesu G, Surabhi RM, Park KJ, Ninomiya-Tsuji J, Matsumoto K (2003). TAK1 is critical for IkappaB kinase-mediated activation of the NF-kappaB pathway.. J Mol Biol.

[32] Omori E, Matsumoto K, Sanjo H, Sato S, Akira S (2006). TAK1 is a master regulator of epidermal homeostasis involving skin inflammation and apoptosis.. J Biol Chem.

[33] Shim JH, Xiao C, Paschal AE, Bailey ST, Rao P (2005). TAK1, but not TAB1 or TAB2, plays an essential role in multiple signaling pathways in vivo.. Genes Dev.

[34] Inagaki M, Omori E, Kim JY, Komatsu Y, Scott G (2008). TAK1-binding protein 1, TAB1, mediates osmotic stress-induced TAK1 activation but is dispensable for TAK1-mediated cytokine signaling.. J Biol Chem.

[35] Li MG, Katsura K, Nomiyama H, Komaki K, Ninomiya-Tsuji J (2003). Regulation of the interleukin-1-induced signaling pathways by a novel member of the protein phosphatase 2C family (PP2Cepsilon).. J Biol Chem.

[36] Baril C, Sahmi M, Ashton-Beaucage D, Stronach B, Therrien M (2009). The PP2C Alphabet Is a Negative Regulator of Stress-Activated Protein Kinase Signaling in Drosophila.. Genetics.

[37] Lu G, Kang YJ, Han J, Herschman HR, Stefani E (2006). TAB-1 modulates intracellular localization of p38 MAP kinase and downstream signaling.. J Biol Chem.

[38] Askari N, Beenstock J, Livnah O, Engelberg D (2009). p38 is Active in vitro and in vivo when Monophosphorylated on Thr180.. Biochemistry.

[39] Ben Levy R, Hooper S, Wilson R, Paterson HF, Marshall CJ (1998). Nuclear export of the stress-activated protein kinase p38 mediated by its substrate MAPKAP kinase-2.. Curr Biol.

[40] Bertelsen M, Sanfridson A (2007). TAB1 modulates IL-1alpha mediated cytokine secretion but is dispensable for TAK1 activation.. Cell Signal.

[41] Casola A, Henderson A, Liu T, Garofalo RP, Brasier AR (2002). Regulation of RANTES promoter activation in alveolar epithelial cells after cytokine stimulation.. Am J Physiol Lung Cell Mol Physiol.

[42] Prickett TD, Ninomiya-Tsuji J, Broglie P, Muratore TL, Shabanowitz J (2008). TAB4 stimulates TAK1-TAB1 phosphorylation and binds polyubiquitin to direct signaling to NF-kappa B.. J Biol Chem.

[43] Mendoza H, Campbell DG, Burness K, Hastie J, Ronkina N (2008). Roles for TAB1 in regulating the IL-1-dependent phosphorylation of the TAB3 regulatory subunit and activity of the TAK1 complex.. Biochem J.

[44] Engel K, Kotlyarov A, Gaestel M (1998). Leptomycin B-sensitive nuclear export of MAPKAP kinase 2 is regulated by phosphorylation.. EMBO J.

[45] Lu M, Lin SC, Huang Y, Kang YJ, Rich R (2007). XIAP induces NF-kappaB activation via the BIR1/TAB1 interaction and BIR1 dimerization.. Mol Cell.

[46] Brown K, Vial SC, Dedi N, Long JM, Dunster NJ (2005). Structural basis for the interaction of TAK1 kinase with its activating protein TAB1.. J Mol Biol.

[47] Dingwall C, Laskey RA (1998). Nuclear import: a tale of two sites.. Curr Biol.

[48] Chuderland D, Konson A, Seger R (2008). Identification and characterization of a general nuclear translocation signal in signaling proteins.. Mol Cell.

[49] Thiefes A, Wolter S, Mushinski JF, Hoffmann E, Dittrich-Breiholz O (2005). Simultaneous blockade of NFkappaB, JNK, and p38 MAPK by a kinase-inactive mutant of the protein kinase TAK1 sensitizes cells to apoptosis and affects a distinct spectrum of tumor necrosis target genes.. J Biol Chem.

[50] Holtmann H, Winzen R, Holland P, Eickemeier S, Hoffmann E (1999). Induction of interleukin-8 synthesis integrates effects on transcription and mRNA degradation from at least three different cytokine- or stress-activated signal transduction pathways.. Mol Cell Biol.

[51] Winzen R, Kracht M, Ritter B, Wilhelm A, Chen CY (1999). The p38 MAP kinase pathway signals for cytokine-induced mRNA stabilization via MAP kinase-activated protein kinase 2 and an AU-rich region-targeted mechanism.. EMBO J.

[52] Makarova O, Kamberov E, Margolis B (2000). Generation of deletion and point mutations with one primer in a single cloning step.. Biotechniques.

[53] Hoffmann E, Thiefes A, Buhrow D, Dittrich-Breiholz O, Schneider H (2005). MEK1-dependent delayed expression of Fos-related antigen-1 counteracts c-Fos and p65 NF-kappaB-mediated interleukin-8 transcription in response to cytokines or growth factors.. J Biol Chem.

[54] Rzeczkowski K, Beuerlein K, Muller H, Dittrich-Breiholz O, Schneider H (2011). c-Jun N-terminal kinase phosphorylates DCP1a to control formation of P bodies.. J Cell Biol.

