# Supplementary figures and images for: Identification and Functional Characterization of Novel Phosphorylation Sites in TAK1-Binding Protein (TAB) 1
Source: PLoS One. 2011 Dec 22;6(12):e29256. doi: 10.1371/journal.pone.0029256 (PMC3245275; doi:10.1371/journal.pone.0029256)

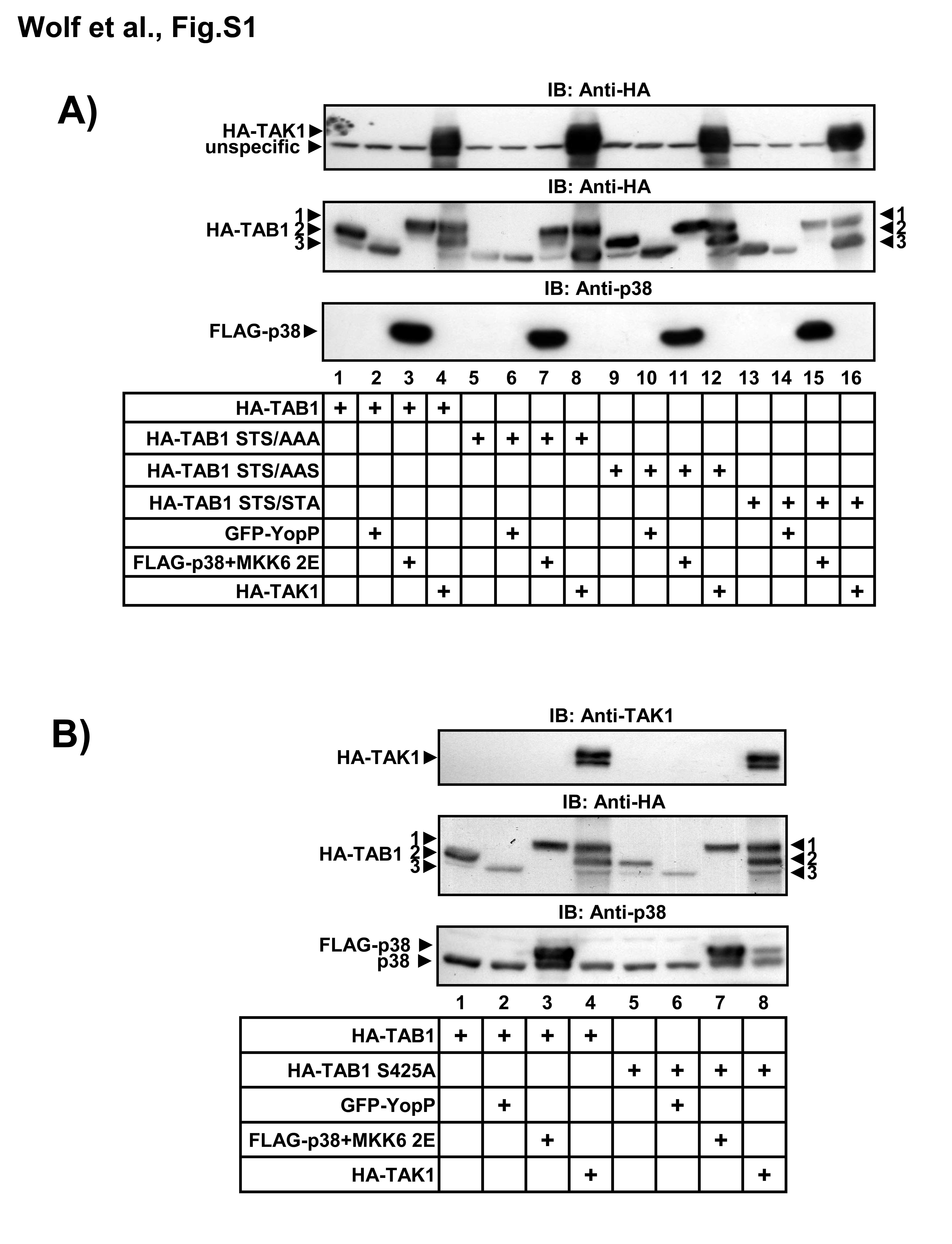

Supplement: Figure S1 — Evidence for new phosphorylation sites in TAB1 in addition to S423, T431 and S438. A) HEK293IL-1R cells were transiently transfected with expression vectors for HA-TAB1 wild type, or versions in which S423/T431/S438 (STS) were mutated to alanine as indicated alone or in combination with GFP-YopP, FLAG-p38 MAPK plus MKK62E or HA-TAK1. B) A similar experiment as in A) was performed using a TAB1 mutant in which S425 was mutated to alanine (HA-TAB1 S425A). 24 h later, cells were lysed followed by immunoblotting (IB) to detect HA-TAK1, FLAG-p38 MAPK or HA-TAB1 using the indicated antibodies. Black arrowheads indicate the three forms of TAB1 (numbered 1–3) with different mobility on SDS-PAGE as previously described by us [15]. Explanation: As shown in Fig. S1A, a retarded mobility form 1 of TAB1 is induced by co-expression of MKK6-p38 MAPK (Fig. S1A, lane 3) or by TAB1-activated TAK1 (Fig. S1A, lane 4). In the absence of stimulation, form 2 shows intermediate mobility and represents a constitutive form of TAB1 which is found in unstimulated cells (Fig. S1A, lane 1). Form 3 of TAB1 displays fastest mobility and is observed by either mutating S438 (Fig. S1A, lane 13) or by intracellular co-expression of the bacterial protease YopP (Fig. S1A, lane 2) which we have shown previously to inhibit the TAK1-MKK6-p38 MAPK pathway [15]. In line with this result, TAB1 form 3 is also found in cells lacking p38α MAPK or treated with SB203580 [15], implying that S438 is constitutively phosphorylated by a low level of active p38 MAPK found in unstimulated cells. However, a version of TAB1 in which S423, T431 and S438 were mutated to alanine was still shifted by MKK6-activated p38 MAPK (Fig. S1A, lane 7) or by TAB1-activated TAK1 (Fig. S1A, lane 8) suggesting that active p38 MAPK and TAK1 phosphorylate novel sites in TAB1 in addition to the three well-characterized S423, T431 and S438 residues. (TIF) [file pone.0029256.s001.tif]

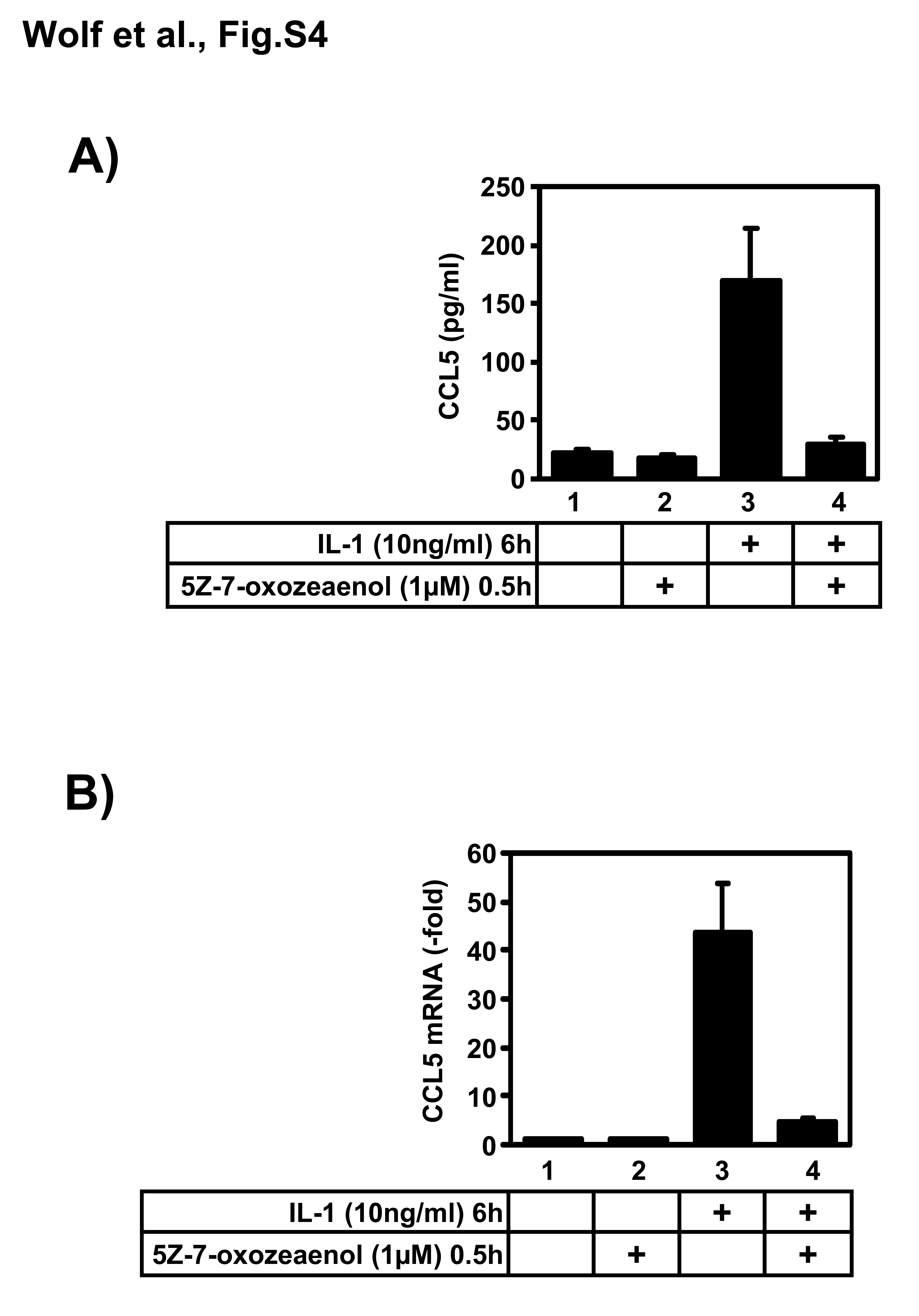

Supplement: Figure S4 — TAK1-dependent activation of the CCL5 gene in A549 cells. A459 lung epithelial carcinoma cells were treated for 30 min with the TAK1 inhibitor 5Z-7-oxozeaenol (1 µM) followed by IL-1α (10 ng/ml) for 6 h, IL-1 alone or were left untreated. Thereafter, CCL5 secretion in the supernatant (A) or CCL5 mRNA expression (B) were determined by specific ELISA and RT-qPCR, respectively. Shown are mean values +/− s.e.m. form 3 independent experiments. (TIF) [file pone.0029256.s004.tif]
